# Adressieren Handlungsempfehlungen der Versorgungspraxis die Stabilität von häuslichen Versorgungsarrangements von Menschen mit Demenz? – Eine Dokumentenanalyse

**DOI:** 10.1007/s00391-022-02024-8

**Published:** 2022-02-01

**Authors:** Iris Hochgraeber, Jan Dreyer, Kerstin Köhler, Christiane Pinkert, Bernhard Holle

**Affiliations:** 1 Deutsches Zentrum für Neurodegenerative Erkrankungen e. V. (DZNE), Witten, Deutschland; 2 Department Pflegewissenschaft, Universität Witten/Herdecke, Witten, Deutschland

**Keywords:** Leitlinie, Informelle Pflege, Pflegearrangement, Versorgungsstrukturen, Pflegende Angehörige, Recommendations for action, Informal care, Family care, Health care structures, Family caregivers

## Abstract

**Hintergrund:**

Die Begleitung von Menschen mit Demenz (MmD) in der Häuslichkeit übernehmen überwiegend Angehörige mit der Intention, die Versorgung so lange wie möglich zu Hause aufrechtzuerhalten. Im DZNE-SoCA-Projekt wurde eine Theorie mittlerer Reichweite zur Stabilität von häuslichen Versorgungsarrangements für MmD (SoCA-Dem-Theorie) entwickelt, um das komplexe Phänomen der Stabilität besser zu verstehen, einen theoretischen Bezugsrahmen für weitere Forschung anzubieten und Orientierung für die (Weiter‑)Entwicklung von Versorgungsstrukturen zu schaffen.

**Ziel der Arbeit:**

Das Ziel dieser Teilstudie des SoCA-Projektes ist es zu prüfen, ob die SoCA-Dem-Theorie in der deutschen Versorgungspraxis handlungsleitend für die Beratung und Begleitung mit versorgenden Angehörigen (vA) von MmD sein kann.

**Material und Methode:**

Es wurden 2 Handlungsempfehlungen für professionelle Akteure im Gesundheitswesen – der „Qualitätsrahmen für Beratung in der Pflege“ des Zentrums für Qualität in der Pflege (ZQP) und die S3-Leitlinie „Pflegende Angehörige von Erwachsenen“ der Deutschen Gesellschaft für Allgemeinmedizin und Familienmedizin e. V. (DEGAM) – inhaltsanalytisch ausgewertet, um Bezugspunkte zur SoCA-Dem-Theorie herauszuarbeiten.

**Ergebnisse:**

Die meisten Konzepte, die die Stabilität der häuslichen Versorgung beeinflussen, werden in beiden Handlungsempfehlungen aufgegriffen. Die SoCA-Dem-Theorie verdeutlicht die Bedeutung des Zusammenspiels der verschiedenen Konzepte; in den Handlungsempfehlungen bleiben diese dynamischen Verbindungen unklar.

**Schlussfolgerung:**

Die SoCA-Dem-Theorie ist anschlussfähig an den deutschen Versorgungskontext und kann zukünftig dahingehend unterstützen, den Blick von einer eher belastungsorientierten Sicht auf Versorgung hin zu einer umfassenden Betrachtung der Situation zu wenden.

**Zusatzmaterial online:**

Zusätzliche Informationen sind in der Online-Version dieses Artikels (10.1007/s00391-022-02024-8) enthalten.

## Abstract (en)

**Background:**

The care of people with dementia (PwD) living at home is mainly provided by family carers who intend to maintain care at home for as long as possible. In the DZNE-SoCA project, a middle range theory of stability of home-based care arrangements for people living with dementia (SoCA-Dem theory) has been developed. The theory helps to understand the complex phenomenon of stability, provides a theoretical framework that can guide future research and can be used for the (further) development of home care structures.

**Aim:**

The aim of this substudy of the SoCA project was to examine whether the SoCA-Dem theory can guide German health and social care practice in dealing with family carers of PwD.

**Material and methods:**

The two guidelines for healthcare professionals, the concept of the Centre for Quality in Care (ZQP) “Quality framework for counselling in care” and the DEGAM guidelines for general practitioners “Family carers of adults”, were evaluated using a content analysis with respect to the SoCA-Dem theory.

**Results:**

Most concepts that constitute stability are addressed in both guidelines. The SoCA-Dem theory illustrates the importance of the interaction between the different concepts for the stability of home-based care arrangements. In the guidelines, the dynamic interplay remains unclear.

**Conclusion:**

The SoCA-Dem theory seems to be compatible with the German health care context and can support a future shift from a stress-oriented view of giving care to a more comprehensive one.

Die meisten Menschen mit Demenz leben zu Hause und werden von ihren Angehörigen mit oder ohne externe Unterstützung versorgt. Angehörige wünschen sich, die Versorgung so lange wie möglich zu Hause fortzusetzen, und sind dabei vor große Herausforderungen gestellt.

## Hintergrund und Zielsetzung

Die Versorgung eines Menschen mit Demenz (MmD) in der eigenen Häuslichkeit muss nicht zwingend mit negativen Folgen für den versorgenden Angehörigen (vA) assoziiert sein, sondern wird von vielen vA durchaus auch positiv empfunden [15]. Im Verlauf einer Demenz gibt es immer wieder Veränderungen, auf die vA reagieren müssen und die zu einem Abbruch der häuslichen Versorgung führen können [14]. Bei der Aufrechterhaltung der häuslichen Versorgung spielt die Stabilität des Versorgungsarrangements eine zentrale Rolle [12].

Bei der Stabilisierung des Versorgungsarrangements kommt der Beratung bei schwierigen Versorgungssituationen und bei der Auswahl passender Angebote eine wichtige Rolle zu. In der deutschen Pflegeversicherung gibt es die Pflicht der Leistungsanbieter zur Beratung (§§ 7, 7a, 27, 373, 45 SGB XI) [2]. Hausärzt*innen wiederum sind zentrale Ansprechpartner*innen für vA [1] und die wichtigste Berufsgruppe, wenn es darum geht, den Zugang zu Hilfsangeboten zu ermöglichen [1, 5]. Jedoch fühlen sich Hausärzt*innen häufig unsicher, welche Rolle sie bei der Versorgung eines MmD und damit auch im Umgang mit den vA spielen [11]. Seit 2018 werden sie von einer S3-Leitlinie der DEGAM zum Umgang mit „pflegenden Angehörigen von Erwachsenen“ (im Folgenden DEGAM-Leitlinie genannt darin unterstützt [7]. Pflegeberatungsstellen nehmen ebenfalls eine wichtige Funktion in der Gestaltung der häuslichen Versorgung eines MmD ein. Das Zentrum für Qualität in der Pflege (ZQP) hat 2016 einen Qualitätsrahmen für Beratung in der Pflege (im Folgenden ZQP-Empfehlung genannt) veröffentlicht [16], der vA in den Fokus nimmt und zur Stabilisierung häuslicher Versorgungsarrangements beitragen will.

In der nationalen und internationalen Literatur wird jedoch bis dato wenig reflektiert, was hinter dem Phänomen der Stabilität steht, und welche Faktoren sie beeinflussen. Im Projekt „*S*tability *o*f home-based *c*are *a*rrangements for people living with dementia“ (SoCA) des Deutschen Zentrums für Neurodegenerative Erkrankungen e. V. (DZNE) am Standort Witten wurden eine Arbeitsdefinition [13] und eine Theorie mittlerer Reichweite zur Stabilität in häuslichen Versorgungsarrangements für MmD [6] entwickelt. Stabilität wird als dynamischer Prozess über den gesamten Versorgungsverlauf konzipiert, der durch das Handeln der vA, die auf Veränderungen der Situation reagieren müssen, gestaltet wird. Dieses Ausbalancieren der vA wird durch die Rolle als vA, die zur Verfügung stehenden Ressourcen und die dyadische Beziehung zwischen MmD und vA beeinflusst. Dabei kann Stabilität nur entstehen, wenn die Bedürfnisse der Beteiligten berücksichtigt werden. Eingebettet ist dieser Prozess in das Gesundheitswesen und die Kultur und Gesellschaft.

### SoCA-Dem-Theorie mittlerer Reichweite

**Figure 1:**
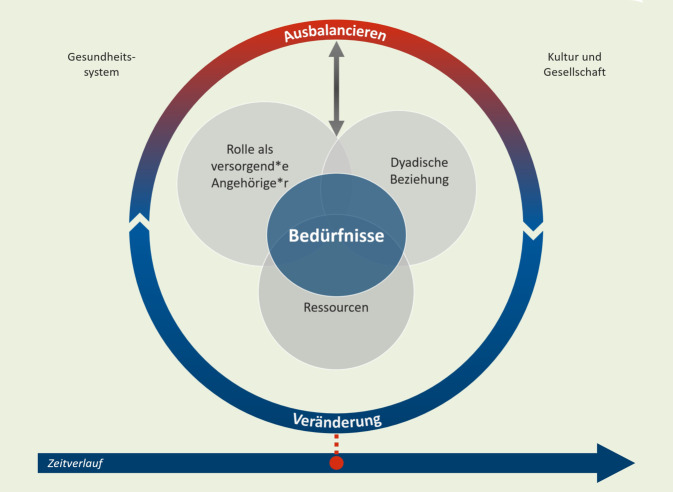

Die SoCA-Dem-Theorie wurden in einer Metastudie auf Basis einer aufwendigen Literaturanalyse entwickelt und in einem konzeptionellen Modell visualisiert, das 3 Komponenten beinhaltet, die sich gegenseitig beeinflussen (Abb. 1; [6]).

#### Komponenten

##### Komponente 1.

Das *Trajekt* beschreibt den zeitlichen Verlauf der Demenz und der Versorgung (Abb. 1*Pfeil unten*) sowie die Handlungen der vA (Abb. 1*Kreis*). Die Situation des Versorgungsarrangements stellt sich am Anfang des Trajektes anders dar als in einer späten Phase, bei einer palliativen Versorgung oder einem Umzug in eine Pflegeeinrichtung. Es treten also im Verlauf vorhersehbare sowie unerwartete Veränderungen auf. Darauf haben die Angehörigen zu reagieren, um die Versorgungssituation stabil zu halten. Dieses Handeln ist als Ausbalancieren charakterisiert. Ausbalancieren kann sowohl ein innerer Prozess (z. B. eine Haltungsänderung) als auch eine praktische Handlung sein. Instabilität tritt auf, wenn eine Veränderung zu einer Krise führt und es den versorgenden Angehörigen nicht (mehr) gelingt, die Situation entsprechend anzupassen.

##### Komponente 2.

Die *Charakteristika* des Versorgungsarrangements, die in der in der Mitte des Modells (Abb. 1) dargestellt sind, zeigen in der Zusammenschau die Konzepte, die die Stabilität von häuslichen Versorgungsarrangements prägen. Das zentrale Konzept in der Mitte sind die Bedürfnisse der Menschen mit Demenz und der vA. Die Theorie geht davon aus, dass eine Adressierung der Bedürfnisse Voraussetzung für eine stabile Situation in der häuslichen Versorgung ist. Ein weiteres Konzept ist die Rolle als versorgende*r Angehörige*r. Der Übergang in diese Rolle erfolgt in der Regel schrittweise und z. T. unbewusst. Dabei unterscheiden sich die Motive für die Übernahme der Rolle: Manche Angehörige übernehmen diese Aufgabe freiwillig, mit hoher intrinsischer Motivation; andere Angehörige sehen sich durch gesellschaftlich geprägte Erwartungen, Pflichtgefühl und Druck von außen in diese Rolle gedrängt. Das Konzept dyadische Beziehung spielt ebenfalls eine wichtige Rolle. Wenn es den Angehörigen gemeinsam mit dem Menschen mit Demenz gelingt, eine gute, d. h. reziproke Beziehung aufrechtzuerhalten, kann die Versorgung zu Hause eher stabil weiter erfolgen. Eine schlechte Beziehung erhöht hingegen das Belastungsempfinden der Angehörigen und führt u. U. zur Instabilität. Einfluss auf Stabilität hat auch das Konzept Ressourcen. Hierunter fallen zuerst individuelle Ressourcen der Angehörigen (z. B. Resilienz, praktische Fertigkeiten). Weitere Ressourcen sind die informelle Unterstützung durch andere Familienangehörige oder soziale Netzwerke, aber auch die professionelle Unterstützung durch Dienstleister.

##### Komponente 3.

Der *Kontext* adressiert die gesellschaftlichen Rahmenbedingungen, in die die beschriebenen Konzepte eingebettet sind. Gesellschaft und Kultur prägen, wie Angehörige sich selbst und die Stabilität ihres Arrangements wahrnehmen, bzw. was von ihnen erwartet wird. So fühlen sich z. B. versorgende Ehepartner*innen häufig an ihr Eheversprechen gebunden; von Frauen wird meist erwartet, die mit dem weiblichen Geschlecht assoziierte Sorgearbeit innerhalb der Familie zu übernehmen. Das Gesundheitssystem hat ebenfalls Einfluss: Die Verfügbarkeit, gute Zugänglichkeit und Finanzierung von Leistungsangeboten beeinflusst die Angehörigen in ihren Entscheidungen und Handlungen. So kann ein Mangel an passenden und/oder finanzierbaren Angeboten zu einer Gefahr für die Stabilität werden.

Das Ziel dieser Teilstudie des SoCA-Projektes besteht darin zu prüfen, ob erstens die Konzepte der SoCA-Dem-Theorie in handlungsleitenden Dokumenten der Beratung durch Hausärzt*innen und Pflegeberater*innen adressiert werden, und zweitens, wenn sie adressiert werden, wie auf sie Bezug genommen wird. Dafür wurden der „Qualitätsrahmen für Beratung in der Pflege“ des Zentrums für Qualität in der Pflege (ZQP) [16] und die S3-Leitlinie für Hausärzte „Pflegende Angehörige von Erwachsenen“ der Deutschen Gesellschaft für Allgemeinmedizin und Familienmedizin e. V. (DEGAM) [7] auf vorhandene Bezüge zum Phänomen der Stabilität geprüft und möglicher Entwicklungsbedarf formuliert.

## Methoden

Da Hausärzt*innen wichtige Kontaktpersonen für MmD und ihre vA sind und auch professionelle Pflegekräfte und andere Professionen an zahlreichen Stellen eine zentrale und beratende Funktion haben, wurden gezielt die oben genannte DEGAM-Leitlinie sowie die ZQP-Empfehlung ausgewählt und mit Blick auf die Stabilität analysiert. Beide Dokumente sind nicht demenzspezifisch. Die DEGAM-Leitlinie bezieht sich jedoch häufig explizit auf vA von MmD, und auch Teile ihrer zugrunde liegenden Evidenz sind demenzspezifisch.

Die Analyse der Inhalte erfolgte inhaltsanalytisch angelehnt an Schreier [10]. Hier wurde zunächst ein Kategoriensystem deduktiv aus den Konzepten der SoCA-Dem-Theorie abgeleitet. Beim Codieren der beiden Dokumente wurden induktiv neue Unterkategorien hinzugefügt. Anschließend wurden einzelne Codes beschrieben und interpretiert. Diese Analyseschritte wurden wiederholt im SoCA-Team diskutiert und die Ergebnisse interpretiert.

## Ergebnisse

Da die inhaltliche Gestaltung von Handlungsempfehlungen wesentlich von ihrer Zielsetzung abhängt, werden zunächst die Ziele der analysierten Dokumente dargestellt und anschließend die Ergebnisse anhand der Kategorien der SoCA-Dem-Theorie beschrieben (Zusatzmaterial online: Supplement 1).

Die Ziele der beiden untersuchten Dokumente unterscheiden sich deutlich voneinander. So zielt die DEGAM-Leitlinie darauf ab, Hausärzt*innen in der Begleitung eines vA zu unterstützen und dabei u. a. präventive Beratung zu pflegeentlastenden bzw. -unterstützenden Maßnahmen anzubieten sowie auch nichthausärztliche Unterstützungsangebote zu vermitteln (DEGAM S. 11). Die ZQP-Empfehlung hat das explizite Ziel, die Situation der vA und pflegebedürftigen Menschen in einer Beratung durch Berater*innen zu stabilisieren und ihre Versorgungsprobleme konkret zu lösen (ZQP S. 3). In beiden Handlungsempfehlungen stehen die häusliche Versorgung und die Unterstützung der vA im Mittelpunkt. Die DEGAM-Leitlinie setzt dabei einen speziellen Fokus auf das Wohlbefinden bzw. die Gesundheit der vA; die ZQP-Empfehlung hebt die Bewältigung der gesamten Pflegesituation heraus.

### Trajekt

Das Konzept *Veränderung *wird in der ZQP-Empfehlung allgemein als Herausforderung für alle Beteiligten, hauptsächlich für die Hauptpflegeperson, (ZQP S. 5) betrachtet. So haben die vA auf veränderte Situationen zu reagieren. Sollten die vA die Herausforderung nicht selbst bewältigen können, so sollten sie Hilfe von außen in Anspruch zu nehmen (ZQP S. 15). Die DEGAM-Leitlinie fokussiert auf gesundheitliche Veränderungen der vA und der MmD. Da vA ein höheres Risiko für gesundheitliche Einschränkungen (DEGAM S. 26) haben als die Allgemeinbevölkerung und vA von MmD an größerer Erschöpfung und mehr Angststörungen (DEGAM S. 26, 27) leiden, gilt es, dieser Personengruppe vonseiten der Hausarzt*innen besondere Beachtung zu schenken (DEGAM S. 34). So bilden Veränderungen, beim vA (z. B. gesundheitliche Einschränkungen) oder beim MmD (z. B. schlechtere Körperhygiene oder Verhaltensveränderungen) oder bei beiden (z. B. Veränderung der Beziehung) oder eine allgemeine Veränderung der Pflegesituation (DEGAM S. 55) für Hausärzt*innen immer einen Anlass für ein Gespräch und eine Anamnese. Veränderungen treten im Trajekt eines Versorgungsarrangements immer wieder auf. Für die professionellen Akteure bedeutet dies einzuschätzen, an welchen Stellen vA Unterstützung benötigen.

Das Konzept *Ausbalancieren *erscheint in beiden Dokumenten nur indirekt. Es wird darauf hingewiesen, dass vA vielfältig praktisch handeln (DEGAM S. 25), um ihre und die Bedürfnisse der MmD zu erfüllen. Diesem Handeln geht häufig das Treffen von Entscheidungen voraus (ZQP S. 3). Vor einer Entscheidung steht häufig ein Abwägungsprozess, bei dem mögliche Szenarien, Vor- und Nachteile, aber auch Konsequenzen eingeschätzt werden. Hausärzt*innen und Pflegeberater*innen können eingreifen, um die Handlungsfähigkeit der vA wiederherzustellen und in verschiedenen Bereichen (z. B. Ressourcen) zu unterstützen (ZQP S. 7). Somit soll professionelle Beratung das Ausbalancieren der vA unterstützen. Ebenfalls sollen Gespräche zur Lösungsfindung beitragen (ZQP S. 12, DEGAM S. 55).

### Charakteristika

Das Konzept* Bedürfnisse *erscheint in der ZQP-Empfehlung, wenn es um konkrete Anlässe für Beratung geht oder der Hinweis darauf erfolgt, dass die Beratung an den individuellen Hilfebedarfen zu orientieren ist. In der DEGAM-Leitlinie wird der Fokus bei der Empfehlung von Unterstützungsangeboten ebenfalls auf die Bedürfnisse der vA ausgerichtet. In beiden Fällen sind eine Identifizierung und Analyse der Bedürfnisse grundlegend. Inhaltlich werden in der DEGAM-Leitlinie wichtige Bedürfnisse der vA benannt, die häufigsten sind demnach der Bedarf an Information und Unterstützung. Insgesamt ist festzustellen, dass in beiden Dokumenten fast ausschließlich die Bedürfnisse der vA angesprochen werden. Lediglich in der DEGAM-Leitlinie wird darauf hingewiesen, dass die Ursache von herausforderndem Verhalten eines MmD u. a. in seinen unerfüllten Bedürfnissen liegen kann (DEGAM S. 56).

In beiden Dokumenten wird die Rolle als versorgende*r Angehörige*r als wichtiger Aspekt verstanden, der sowohl positive als auch negative Folgen für vA haben kann. Unerwartete und schnell erforderliche Übernahmen werden in der DEGAM-Leitlinie (DEGAM S. 10) als problematisch eingeschätzt, während ein gutes Entlassungsmanagement aus einem Krankenhaus und eine geplante Übernahme der Rolle als vA hilft, sich in der neuen Rolle zu finden (DEGAM S. 61). Prominent hervorgehoben wird in beiden Dokumenten die Belastung der vA. Diese kann zu Überforderung und gesundheitlichen Folgen (ZQP S. 3), aber auch zu sozialer Isolation führen (DEGAM S. 29). Sie ist häufig mit Rollenkonflikten im Zusammenhang mit der Vereinbarkeit von Pflege und Beruf (DEGAM S. 29) oder der Familie (DEGAM S. 38) assoziiert. In beiden Dokumenten werden auch positive Aspekte des Versorgens genannt. Diese sind z. B. ein Gefühl von Sinnhaftigkeit, Zufriedenheit oder ein persönlicher Gewinn durch die Pflegetätigkeit (DEGAM S. 4; ZQP S. 6). Es gilt, die positiven Erfahrungen in Gesprächen zu thematisieren und Wertschätzung zu vermitteln (DEGAM S. 47).

Das Konzept der *dyadischen Beziehung* wird in der DEGAM-Leitlinie als relevanter Aspekt gesehen. Die Qualität der Beziehung oder auch der Verlust hat Einfluss auf die Belastung (DEGAM S. 28; S. 29; S. 46). So stellt eine Veränderung in der Beziehung einen Anlass für die Hausärzt*innen dar, ein Gespräch zu führen (DEGAM S. 34), diesen Umstand genauer zu betrachten und evtl. eine Familienkonferenz einzuberufen (DEGAM S. 38). Auch strukturelle Aspekte einer Beziehung, wie die Wohnsituation oder die Entfernung zwischen den Haushalten, (DEGAM S. 38) müssen bedacht werden. In der ZQP-Empfehlung wird die dyadische Beziehung nicht erwähnt.

Das Konzept* Ressourcen* wird in beiden Dokumenten aufgegriffen. Insgesamt werden vorhandene, aber auch mangelnde oder ungenutzte Ressourcen (ZQP S. 6) als Anlass für eine Beratung gesehen (ZQP S. 3). Hier sollen dann unterschiedliche Ressourcen gestärkt werden, z. B. durch Wissensvermittlung (ZQP S. 14) oder die Vermittlung praktischer Fähigkeiten (ZQP S. 14). Auch die finanziellen Ressourcen bzw. die Finanzierung der Versorgung durch die Pflegeversicherung sollen in Gesprächen ein Thema sein (DEGAM S. 38; ZQP, S. 12). Das informelle Netzwerk spielt als psychosoziale Unterstützung eine Rolle (ZQP S. 12; DEGAM S. 41). In beiden Dokumenten liegt der Schwerpunkt aber auf den formalen Ressourcen in Form von zu vermittelnden passenden Unterstützungsangeboten und Hilfsmitteln (z. B. DEGAM S. 11; ZQP S. 3). Die Beziehung zwischen Hausärzt*innen bzw. Berater*innen und den vA scheint eine wichtige Funktion zu erfüllen. Der ZQP-Empfehlung zufolge ist für den Aufbau eines Vertrauensverhältnisses sowohl Empathie als auch Fachkompetenz notwendig (ZQP S. 16). Für das Verhältnis zwischen Hausärzt*innen und vA ist die Kommunikation wichtig, diese gilt es, an den Bedürfnissen der vA zu orientieren (DEGAM S. 30).

### Kontext

Auf den Kontextaspekt der* Kultur und Gesellschaft *wird in beiden Dokumenten am Rande eingegangen. Beratung soll sich am kulturellen Hintergrund und der Lebenswelt der Ratsuchenden orientieren (ZQP S. 26; DEGAM S. 31) und diese verstehen. Auch Angebote können am kulturellen Hintergrund der Betroffenen ausgerichtet sein (ZQP S. 7).

Der Kontextaspekt *Gesundheitssystem* wird aufgegriffen. Hier wird darauf eingegangen, dass bestimmte Angebote im deutschen Gesundheitssystem nicht oder nicht regelhaft angeboten und finanziert werden (z. B. multimodale Interventionen) (DEGAM S. 70). Ebenfalls wird die gesetzliche Grundlage erwähnt. So haben vA die Möglichkeit, spezielle Rehabilitations- und Vorsorgemaßnahmen in Anspruch zu nehmen (DEGAM S. 83). Die Umsetzung der Beratung nach § 7a SGB XI ist sehr unterschiedlich und nicht für alle Personen gewährleistet (ZQP S. 4). Weiterhin ist die Nutzung der Beratungsangebote, die laut Gesetz möglich sind, gering (ZQP S. 4).

## Diskussion

Die Analyse der ZQP-Empfehlung und der DEGAM-Leitlinie zeigt, dass beiden Dokumenten ein komplexes Verständnis von der Versorgung eines Menschen (mit Demenz) in der Häuslichkeit zugrunde liegt. Die in der SoCA-Dem-Theorie beschriebenen Konzepte finden sich auch in den beiden analysierten Dokumenten wieder. Lediglich in der ZQP-Empfehlung gibt es keine Aussagen zur dyadischen Beziehung. Kritisch anzumerken ist, dass die Dokumente bisher nicht auf Verbindungen zwischen den Konzepten hinweisen. Die SoCA-Dem-Theorie bietet eine theoretische Grundlage, welche Konzepte für stabile Versorgungsarrangements von Relevanz sind, und wie die Verbindungen zwischen diesen Konzepten aussehen. Wenngleich einschränkend anzumerken ist, dass das SoCA-Dem-Team diese Verbindungen zum jetzigen Zeitpunkt noch nicht vollständig ausgearbeitet hat. Es wird davon ausgegangen, dass ein detailliertes Verständnis von der Gestaltung häuslicher Versorgungsarrangements dazu beitragen kann, die Versorgung zu Hause nachhaltig zu verbessern.

In den analysierten Dokumenten wird deutlich, dass die Adressaten von Beratung klar die vA sind und nicht die MmD selbst. Die SoCA-Dem-Theorie bietet erste Hinweise, dass mit Blick auf die Stabilität von häuslichen Versorgungsarrangements eine Adressierung der Bedürfnisse aller Beteiligten notwendig ist [13] und die MmD keinesfalls nur als belastende Faktoren für die vA gesehen werden können. Zur Umsetzung dieser Forderung bedarf es Forschung, die auch die Sichtweise der MmD in den Blick nimmt. Aktuelle Entwicklungen gehen jedoch über den reinen Einbezug von MmD als Studienteilnehmer*innen hinaus und wollen MmD mit einem partizipativen Ansatz oder als Koforschende in Projekte involvieren [4]. Auch in der Entwicklung von Handlungsempfehlungen und Leitlinien gibt es das Bestreben, die Betroffenen selbst und ihre Perspektive einzubeziehen [8].

Beide analysierte Dokumente haben einen vorwiegend stresstheoretischen Blick auf die Situation eines häuslichen Versorgungsarrangements und rücken damit die Belastung der vA in den Fokus. Das Belastungserleben zu reduzieren und die Analyse der auslösenden Faktoren sind zweifellos wichtige Aspekte. Die SoCA-Dem-Theorie regt darüber hinaus dazu an, weitere Aspekte, die für die Stabilität des Versorgungsarrangements bedeutend sind, in den Blick zu nehmen: Positive oder gewinnbringende Aspekte von Versorgung sind nicht gegenteilig zu den negativen Folgen, sondern können durchaus gleichzeitig in hohem Maße auftreten und sollten gestärkt werden [9]. Frewer-Graumann [3] spricht von Selbstfürsorgeelementen, die in den Alltag der vA zu integrieren sind, um die Stabilität eines Arrangements zu erhalten. Um diese Selbstfürsorge zu gewährleisten, braucht es Unterstützungsleistungen von außen. Zur Inanspruchnahme dieser Leistungen und dafür, dass vA diese Leistungen als hilfreich wahrnehmen, sieht Frewer-Graumann [3] die Einigung auf eine gemeinsame Situationsdefinition und damit die Haltung der Unterstützer*innen als eine wichtige Voraussetzung. In der Arbeit mit vA reicht es also nicht, nur an den belastenden bzw. entlastenden Faktoren anzusetzen. Vielmehr ist die komplexe häusliche Situation mit allen Facetten zu analysieren und bei Einbezug von Unterstützer*innen von außen auf deren Haltung zu achten. Dabei ist es von besonderer Bedeutung, die Rolle des vA, die dyadische Beziehung, die Ressourcen und die Bedürfnisse des MmD und des vA im Blick zu haben und ebenso die Verbindungen dieser Konzepte zueinander zu kennen, um so ein individuelles Bild bezüglich der Stabilität eines einzelnen Versorgungsarrangements zu gewinnen.

Für zukünftige Forschung zu häuslichen Versorgungsarrangements mit Menschen mit Demenz oder auch bei der Entwicklung von Leitlinien kann die SoCA-Dem-Theorie als theoretischer Bezugsrahmen dienen. Allerdings gilt es gleichzeitig, die Theorie empirisch zu überprüfen und ggf. fortlaufend weiterzuentwickeln.

## Fazit

- Versorgende Angehörige (vA) sind häufig die wichtigsten Personen in der häuslichen Versorgung von Menschen mit Demenz (MmD), und es gilt, sie durch alle Akteure im Gesundheitswesen zu unterstützen.
- Die DEGAM-Leitlinie und die ZQP-Empfehlung greifen die komplexe Situation einer häuslichen Versorgung umfangreich auf.
- Die Versorgung durch Hausärzt*innen und die Beratung durch Pflegefachkräfte ist individuell und an die spezielle Situation eines vA anzupassen.
- Um die Stabilität eines häuslichen Versorgungsarrangements zu unterstützen, sind ein komplexes Verständnis der Situation und das Wissen um die Bedeutung der stabilitätbeeinflussenden Faktoren hilfreich. Hierfür sollten die Verbindungen der Konzepte zukünftig detaillierter untersucht werden.
- In zukünftigen Handlungsempfehlungen zum Umgang mit vA gilt es, weg von der Belastungsorientierung hin zu einem Fokus auf Stabilität sowie die Stabilität fördernden Aspekten zu kommen und eine gemeinsame Betrachtung aller Aspekte und ihrer Verbindungen anzustreben.

## Supplementary Information





