# Supplementary material for: Do guidelines for care practice address the stability of home-based care arrangements for people with dementia?—A document analysis
Source: Z Gerontol Geriatr. 2022 Feb 1;56(3):209–14. [Article in German] doi: 10.1007/s00391-022-02024-8 (PMC10191907; doi:10.1007/s00391-022-02024-8)
Supplement: Supplementary file 1 [file 391_2022_2024_MOESM1_ESM.docx]

**Supplement 1**

*SoCA-Dem Theorie* mittlerer Reichweite

Die SoCA-Dem Theorie wurden in einer Meta-Studie auf Basis einer aufwendigen Literaturanalyse entwickelt und in einem konzeptionellen Modell visualisiert, das drei Komponenten beinhaltet, die sich gegenseitig beeinflussen (siehe Abb. 1) [1].
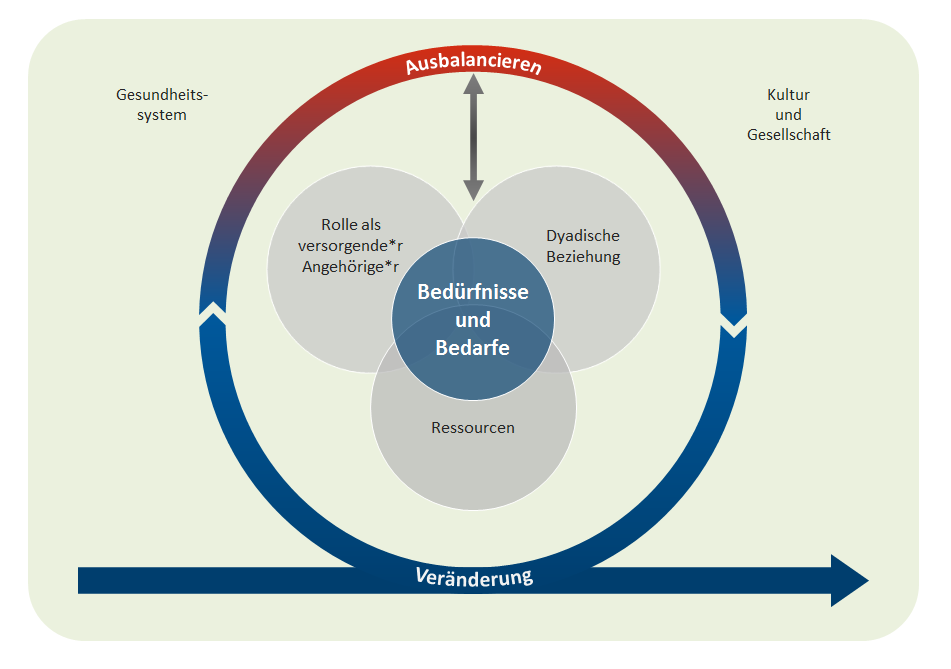


**Abb. 1: Konzeptionelles Modell der Stabilität von häuslichen Versorgungsarrangements für MmD (SoCA-Dem) (Übersetzung durch das SoCA Team auf Basis [1])**

Komponenten:

- Komponente 1: das **Trajekt** beschreibt den zeitlichen *Verlauf* der Demenz und der Versorgung (Pfeil unten) sowie die Handlungen der vA (Kreis). Die Situation des Versorgungsarrangements stellt sich am Anfang des Trajektes anders dar, als in einer späten Phase, bei einer palliativen Versorgung oder einem Umzug in eine Pflegeeinrichtung. Es treten also im Verlauf vorhersehbare sowie unerwartete *Veränderungen* auf. Darauf haben die Angehörigen zu reagieren, um die Versorgungssituation stabil zu halten. Dieses Handeln ist als *Ausbalancieren* charakterisiert. Ausbalancieren kann sowohl ein innerer Prozess (z. B. eine Haltungsänderung) als auch eine praktische Handlung sein. Instabilität tritt auf, wenn eine Veränderung zu einer Krise führt und es den versorgenden Angehörigen nicht (mehr) gelingt, die Situation entsprechend anzupassen.
- Komponente 2: die **Charakteristika** des Versorgungsarrangements, die in der in der Mitte des Modells dargestellt ist, zeigen in der Zusammenschau die Konzepte, die die Stabilität von häuslichen Versorgungsarrangements prägen. Das zentrale Konzept in der Mitte sind die *Bedürfnisse* der Menschen mit Demenz und der versorgenden Angehörigen. Die Theorie geht davon aus, dass eine Adressierung der Bedürfnisse Voraussetzung für eine stabile Situation in der häuslichen Versorgung ist. Ein weiteres Konzept ist die *Rolle als versorgende*r Angehörige*r*. Der Übergang in diese Rolle erfolgt in der Regel schrittweise und zum Teil unbewusst. Dabei unterscheiden sich die Motive für die Übernahme der Rolle: Manche Angehörige übernehmen diese Aufgabe freiwillig, mit hoher intrinsischer Motivation; andere Angehörige sehen sich durch gesellschaftlich geprägte Erwartungen, Pflichtgefühl und Druck von außen in diese Rolle gedrängt. Das Konzept *dyadische Beziehung* spielt ebenfalls eine wichtige Rolle. Wenn es den Angehörigen gemeinsam mit dem Menschen mit Demenz gelingt, eine gute, d.h. reziproke Beziehung aufrechtzuerhalten, kann die Versorgung zuhause eher stabil weiter erfolgen. Eine schlechte Beziehung erhöht hingegen das Belastungsempfinden der Angehörigen und führt unter Umständen zu Instabilität. Einfluss auf Stabilität hat auch das Konzept *Ressourcen*. Hierunter fallen zuerst individuelle Ressourcen der Angehörigen (z. B. Resilienz, praktische Fertigkeiten). Weitere Ressourcen sind die informelle Unterstützung durch andere Familienangehörige oder soziale Netzwerke aber auch die professionelle Unterstützung durch Dienstleister.
- Komponente 3: der **Kontext** adressiert die gesellschaftlichen Rahmenbedingungen, in die die beschriebenen Konzepte eingebettet sind. *Gesellschaft und Kultur* prägen, wie Angehörige sich selbst und die Stabilität ihres Arrangements wahrnehmen bzw. was von ihnen erwartet wird. So fühlen sich z. B. versorgende Ehepartner*innen häufig an ihr Eheversprechen gebunden, von Frauen wird meist erwartet, die mit dem weiblichen Geschlecht assoziierte Sorgearbeit innerhalb der Familie zu übernehmen. Das *Gesundheitssystem* hat ebenfalls Einfluss: Die Verfügbarkeit, gute Zugänglichkeit und Finanzierung von Leistungsangeboten beeinflusst die Angehörigen in ihren Entscheidungen und Handlungen. So kann ein Mangel an passenden und/ oder finanzierbaren Angeboten zu einer Gefahr für die Stabilität werden.

**Literatur:**

1. Köhler K, Dreyer J, Hochgraeber I et al. (2021) Towards a middle-range theory of ‘Stability of home-based care arrangements for people living with dementia’ (SoCA-Dem): findings from a meta-study on mixed research. BMJ open 11
