# Supplementary material for: Do guidelines for care practice address the stability of home-based care arrangements for people with dementia?—A document analysis
Source: Z Gerontol Geriatr. 2022 Feb 1;56(3):209–14. [Article in German] doi: 10.1007/s00391-022-02024-8 (PMC10191907; doi:10.1007/s00391-022-02024-8)
Supplement: Supplementary file 2 [file 391_2022_2024_MOESM2_ESM.docx]

**Supplement 2**

| **Tabelle:** Überblick über die Analyseergebnisse | | | |
| --- | --- | --- | --- |
| **Konzepte der SoCA-Dem Theorie** | | **Analyse der ZQP-Empfehlung** | **Analyse der DEGAM-Leitlinie** |
| Trajekt | Veränderung | - Veränderung ist Herausforderung - Angehörige haben darauf zu reagieren | - Gesundheitliche Veränderungen der Angehörigen und der Personen mit Demenz - Veränderungen der Pflegesituation - Gesprächsanlass und Anamnese |
|  | Ausbalancieren | - Praktisch handeln und Entscheidungen treffen - Beratung hilft beim Herstellen der Handlungsfähigkeit | - Vielfältig Handeln - Hausärzt*innen können das Handeln der Angehörigen unterstützen |
| Charakteristika | Bedürfnisse | - Beratungsanlass - Orientierung an Bedürfnissen | - Identifizieren und Analysieren der Bedürfnisse - Orientierung an Bedürfnissen bei Angebotsempfehlung |
|  | Rolle des versorgenden Angehörigen | - Gestaltung der Rollenübernahme - Negative Folgen: Belastung, gesundheitlichen und sozialen Einschränkungen - Positive Folgen: persönliche Entwicklung | - Gestaltung der Rollenübernahme - Negative Folgen: Rollenkonflikte, Belastung, gesundheitlichen und sozialen Einschränkungen - Positive Folgen: Persönlicher Gewinn durch die Pflege |
|  | Dyadische Beziehung |  | - Qualität der Beziehung hat Einfluss auf Belastung - Veränderungen in der Beziehung ist ein Gesprächsanlass |
|  | Ressourcen | - Orientierung an vorhandenen Ressourcen - Mangelnde Ressourcen als Beratungsanlass - Stärkung der Ressourcen - Soziales Netzwerk - Beziehung zum/zur Berater*in | - Mangelnde Ressourcen als Gesprächsanlass - Finanzielle Ressourcen - Soziales Netzwerk - Beziehung zum/zur Hausärzt*in |
| Kontext | Kultur und Gesellschaft | - Orientierung am kulturellen Kontext | - Orientierung am kulturellen Kontext |
|  | Gesundheitssystem | - Beratung ist nicht für alle gleich zugänglich - Geringe Nutzung von Beratung | - Zugänglichkeit von Angeboten stellenweise schlecht |
